# Evidence That Zika Virus Is Transmitted by Breastfeeding to Newborn A129 (*Ifnar1* Knock-Out) Mice and Is Able to Infect and Cross a Tight Monolayer of Human Intestinal Epithelial Cells

**DOI:** 10.3389/fmicb.2020.524678

**Published:** 2020-10-22

**Authors:** Mathieu Hubert, Patricia Jeannin, Julien Burlaud-Gaillard, Philippe Roingeard, Antoine Gessain, Pierre-Emmanuel Ceccaldi, Aurore Vidy

**Affiliations:** ^1^Unité Epidémiologie et Physiopathologie des Virus Oncogènes, Département Virologie, Institut Pasteur, Paris, France; ^2^Unité Epidémiologie et Physiopathologie des Virus Oncogènes, Université de Paris, Paris, France; ^3^Centre National de la Recherche Scientifique, Département Virologie, Institut Pasteur, UMR 3569, Paris, France; ^4^INSERM U1259, Université de Tours et CHU de Tours, Tours, France; ^5^Plate-forme IBiSA de Microscopie Electronique, Université de Tours et CHU de Tours, Tours, France

**Keywords:** Zika virus, mouse model, oral transmission, mother-to-child transmission, breastfeeding, intestinal epithelium, crossing, entry site

## Abstract

Zika virus (ZIKV) belongs to the *Flavivirus* genus in the *Flaviviridae* family. Mainly transmitted via mosquito bites (*Aedes aegypti*, *Aedes albopictus*), ZIKV has been classified in the large category of arthropod-borne viruses, or arboviruses. However, during the past two outbreaks in French Polynesia (2013–2014) and Latin America (2015–2016), several cases of ZIKV human-to-human transmission were reported, either vertically via transplacental route but also horizontally after sexual intercourse. Interestingly, high viral burdens were detected in the colostrum and breast milk of infected women and mother-to-child transmission of ZIKV during breastfeeding was recently highlighted. In a previous study, we highlighted the implication of the mammary epithelium (blood–milk barrier) in ZIKV infectious particles excretion in breast milk. However, mechanisms of their further transmissibility to the newborn via oral route through contaminated breast milk remain unknown. In this study, we provide the first experimental proof-of-concept of the existence of the breastfeeding as a route for mother-to-child transmission of ZIKV and characterized the neonatal oral transmission in a well-established mouse model of ZIKV infection. From a mechanistical point-of-view, we demonstrated for the first time that ZIKV was able to infect and cross an *in vitro* model of tight human intestinal epithelium without altering its barrier integrity, permitting us to consider the gut as an entry site for ZIKV after oral exposure. By combining *in vitro* and *in vivo* experiments, this study strengthens the plausibility of mother-to-child transmission of ZIKV during breastfeeding and helps to better characterize underlying mechanisms, such as the crossing of the newborn intestinal epithelium by ZIKV. As a consequence, these data could serve as a basis for a reflection about the implementation of measures to prevent ZIKV transmission, while keeping in mind breastfeeding-associated benefits.

## Introduction

Zika virus (ZIKV) is a positive single-stranded RNA virus, belonging to the *Flavivirus* genus in the *Flaviviridae* family. It was discovered in 1947 in the Zika forest of Uganda (Dick et al., 1952; Schwartz, 2017) and spread out of Africa, evolving in the African and the Asian lineages. From 1947 to 2007, only a few persons were reported to be infected by ZIKV worldwide. Since no sign of disease was reported, they were suspected to remain asymptomatic or develop mild non-specific symptoms (Musso and Gubler, 2016). However, at the end of the 2000s, the epidemic potential of ZIKV was recognized by its emergence in the Pacific islands, leading to two epidemics in Yap (2007) (Duffy et al., 2009) and in French Polynesia (2013–2014) (Cao-Lormeau et al., 2014; Musso et al., 2018). During the second one, a link between adult ZIKV infection and Guillain-Barré syndrome occurrence was established (Musso et al., 2014; Bautista and Sethi, 2016; Cao-Lormeau et al., 2016). More recently, the virus reached Brazil and spread throughout Latin America in 2015–2016 (Ayllón et al., 2017; Baud et al., 2017; Faria et al., 2017), infecting more than 750,000 persons and resulting in the largest ZIKV outbreak ever reported. During this last outbreak, ZIKV became a public health emergency of international concern (PHEIC) by causing congenital Zika syndromes (CZS) (Driggers et al., 2016; Mlakar et al., 2016; Rasmussen et al., 2016) which were characterized by a large panel of neurological disorders – e.g., microcephaly – in some babies born from infected mothers. Indeed, besides the major importance of mosquito-borne infections, the unexpected number of human-to-human transmissions of the virus constituted one of the major characteristics of this last epidemic. Based on observations, human-to-human transmissions of ZIKV essentially occurred horizontally following sexual intercourses (Musso et al., 2015; D’Ortenzio et al., 2016) and vertically by transplacental route (Grischott et al., 2016). However, because of the rapid increase of the incidence of ZIKV infections, it was difficult to distinguish vector-borne and human-to-human transmissions of ZIKV.

In humans, ZIKV was detected in many body fluids (Grischott et al., 2016; Calvet et al., 2018; Mann et al., 2018) such as urine (Fonseca et al., 2014; Kutsuna et al., 2014; Gourinat et al., 2015; Campos et al., 2016), semen (Mansuy et al., 2016a,b), vaginal secretions (Murray et al., 2017; Penot et al., 2017), saliva (Besnard et al., 2014; Barzon et al., 2016), tears (Tan et al., 2017), nasopharyngeal swabs (Leung et al., 2015), and breast milk (Besnard et al., 2014; Dupont-Rouzeyrol et al., 2016; Cavalcanti et al., 2017; Colt et al., 2017; Sotelo et al., 2017; Mann et al., 2018), raising a number of questions regarding the risk of human-to-human transmission. Particularly, several case studies reported ZIKV RNA shedding as well as infectious particles in the breast milk of infected mothers, with a viral load significantly higher than in serum (Dupont-Rouzeyrol et al., 2016; Cavalcanti et al., 2017; Colt et al., 2017; Sotelo et al., 2017). Moreover, it was shown that the virus persisted in breast milk over 30 days after onset of illness (Sotelo et al., 2017), allowing a sufficient period for mother-to-child transmission (MTCT) via breastfeeding. From an experimental point of view, one study demonstrated also ZIKV presence in the breast milk of infected immunocompromised mice (Regla-Nava et al., 2019), which could be explained by productive infection of both luminal and myoepithelial cells of the mammary epithelium (Hubert et al., 2019). Moreover, post-natal transmission of ZIKV from an infected mother to her breastfed newborn had already been suggested during the 2013–2014 outbreak in French Polynesia (Besnard et al., 2014), but the evidence of MTCT of ZIKV through breast milk was recently highlighted after sequencing of identical isolates from the breast milk of a nursing mother and the plasma of her 5-month-old infant (Blohm et al., 2017, 2018). Given that breast milk serves as a vehicle for MTCT of numerous viruses (Jelliffe and Jelliffe, 1981) such as human immunodeficiency virus – type 1 (HIV-1) (Van de Perre, 2000), human T-cell leukemia virus – type 1 (HTLV-1) (Percher et al., 2016), or cytomegalovirus (CMV) (Numazaki, 1997), and that probable transmissions of some arboviruses (West Nile virus) (Centers for Disease Control and Prevention [CDC], 2002; Hinckley et al., 2007), yellow fever virus (Centers for Disease Control and Prevention [CDC], 2010; Kuhn et al., 2011), dengue virus – serotype 1 (Barthel et al., 2013; Arragain et al., 2017) via breast milk were previously reported, we cannot exclude this body fluid as a serious candidate for oral transmission of ZIKV. Furthermore, ZIKV stability and maintenance of infectivity in breast milk were assessed (Pfaender et al., 2017; Conzelmann et al., 2019), strengthening the potential of breast milk to convey infectious ZIKV during mother-to-child transmission of ZIKV. Finally, two studies demonstrated that non-human primates were susceptible to ZIKV infection, either after oropharyngeal exposure (Newman et al., 2017), or after intra-gastric inoculation (Deng et al., 2017), showing that ZIKV can be orally transmitted. In this study, we addressed the possibility of the early-life oral transmission of ZIKV to the newborn through breastfeeding.

To this end, we assessed a proof-of-concept of the existence of breastfeeding as a plausible route for MTCT of ZIKV. Moreover, because we also showed that neonatal mice were susceptible to ZIKV infection after intragastric inoculation, we wondered whether the intestinal epithelium could serve as portal site for oral transmission of ZIKV. To this end, we showed that different clones of human intestinal epithelial cells were permissive to ZIKV infection, and that ZIKV was basolaterally released after apical infection of an *in vitro* model of a tight intestinal epithelium, without any loss of epithelial integrity.

## Materials and Methods

### Cells

Human enterocyte-like cells Caco-2 (clone 4, Sigma; clone TC7, unreferenced cell line) and HT-29 (ATCC HTB-38) were cultured in DMEM supplemented with L-glutamine, 10% FBS, 10 mM HEPES buffer (Sigma), 100 U/mL penicillin and 100 μg/mL streptomycin. Vero E6 cells (CRL-1586, ATCC) were grown in DMEM supplemented with L-glutamine (Gibco), 10% fetal bovine serum (FBS, Gibco), 100 U/mL penicillin, and 100 μg/mL streptomycin (Gibco). All cells were maintained at 37°C and 5% CO_2_ for culture.

### Establishment of an *in vitro* Model of Intestinal Epithelium

To study ZIKV infection and crossing of the intestinal barrier, we took advantage of an *in vitro* model of intestinal epithelium according to a previous study from our laboratory (Martin-Latil et al., 2012). Briefly, 2.5 × 10^4^ enterocyte-like Caco-2/TC7 cells were seeded onto Transwell inserts (12 mm diameter, 3 μm porosity, polycarbonate, Costar) and grown for at least 14 days, delimiting apical (upper chamber) and basolateral (lower chamber) compartments. Apical and basolateral media were replaced every 2 days, and integrity of Caco-2 monolayers was checked as described below.

### Validation of Monolayer Tightness

Tightness evolution of Caco-2 monolayers was routinely monitored by measuring its *trans*-epithelial electric resistance (TEER; EVOM World Precise Instruments) (Srinivasan et al., 2015). Briefly, electrodes were plunged inside the apical and basolateral compartments of Caco-2-based monolayers cultures, and a continuous current was applied. Resistance of the Caco-2 monolayers was reported every 2 days. After 14 days of culture, permeability of Caco-2 monolayers was also quantified by a fluorimetric method. In detail, basolateral medium was replaced by DMEM without red phenol (Gibco, by Life Technologies) and a FITC-coupled dextran 4 kDa (Sigma) solution was added in the apical compartment. As FITC-Dextran diffused through the monolayer over time, basolateral fluorescence increase and was measured using a fluorescence plate reader (Wallac Victor^2^ and PerkinElmer). Permeability P (cm.min^–1^) of the monolayers was calculated as following:

$$P=\frac{a\times V_{B\,L}}{F\,l\,u\,o_{A\,P}\times F\,d\times S}$$

with “a” the slope (arbitrary units.min^–1^),“V_BL_” the basolateral compartment volume (cm^3^), “Fluo_AP_” the apical inoculum fluorescence (arbitrary units), “Fd” the dilution factor of inoculum, and “S” the filter area (cm^2^). Monolayers for which TEER ≥ 250 Ω.cm^2^ and *p* ≤ 10^–5^ cm.min^1^ were considered as impermeable, according to literature (Martin-Latil et al., 2012). Tightness validation of the model was also completed with morphological integrity study of Caco-2/TC7 monolayers (visualization of tight junctions and brush border) by transmission electron microscopy, as previously described (Monel et al., 2017).

### Mice

Experiments were performed on A129 mice (IFNAR^–/–^) (Muller et al., 1994; Hubert et al., 2019), which were housed and bred in the Institut Pasteur animal facilities accredited by the French Ministry of Agriculture for breeding and performing experiments on live rodents.

### Viruses

Two ZIKV human isolates that belong to the Asian lineage (strain H/PF13, GenBank: KX369547; strain Brazil/2016, GenBank: KU991811) were provided by V. Choumet (Institut Pasteur, Paris, France) and amplified in Vero E6 cells as previously described (Hubert et al., 2019). For viral stocks production, ZIKV suspension was added to Vero E6 cell monolayers at 37°C; 5% CO_2_ for 2 h in DMEM-2% FBS. After adsorption, the inoculum was removed and replaced by fresh DMEM-2% FBS for 3–4 days. After cytopathic effects appearance, supernatants were clarified by centrifugation at 500 *g* for 10 min and aliquoted for −80°C storage until titration by focus forming assay (FFA). Supernatants from cell culture were used as inoculum for cell and animal infections.

### Focus Forming Assay (FFA)

Serial dilutions of the samples were performed in DMEM – 2% FBS. Diluted samples were applied onto 90% confluence Vero cells monolayers for 2 h at 37°C, 5% CO_2_. Inocula were removed after viral adsorption and were replaced by a mixed solution of DMEM – 4% FBS and PBS – 1% carboxymethyl cellulose in a 1:1 ratio. 3 days later, cells were fixated in PBS – 4% paraformaldehyde for 15 min at room temperature (RT), and permeabilized with PBS – 0.1% Triton for 3 min at RT. PBS – 1% bovine serum albumin – 0.1% Tween 20 was applied on cells for 30 min at RT in order to block non- specific antigenic sites. ZIKV envelope protein (E) immune-staining was performed using the mouse anti-E [clone 4G2; home-purified from the ATCC hybridoma (Monel et al., 2017)] antibody as primary antibody and the HRP conjugated goat anti-mouse IgG antibody (Biorad) as secondary. Finally, after addition of freshly prepared peroxidase substrate (Vector Vip; Vector Laboratories) for 5–15 min, purple foci of infection were manually counted.

### Animal Infections

For MTCT experiments, 8–10 weeks-old A129 lactating female mice were intraperitoneally infected with 6,6 × 10^6^ FFU of the American (Brazil/2016) strain of ZIKV at 3 days post-partum. Infection was monitored in mothers’ plasma and MTCT via breastfeeding was monitored in suckling pups’ plasma. Mice were euthanized when weight loss of ≥ 20 or ≥ 10% in association with one suffering symptom were observed.

For oral infection of neonatal mice, A129 females and males were mixed for mating and pregnant mice were housed and bred according to animal welfare recommendations. Different-sized litters were obtained and used as individual groups. Neonatal mice endured two successive viral oral administrations at 2 days-old (50 μL of the inoculum by putting the micropipette tip into the pup suckling mouth) and 5 days-old (intragastric inoculation of 100 μL of the inoculum by using small flexible gastric tubes) with 1 × 10^5^ and 2 × 10^5^ FFU of the American (Brazil/2016) strain of ZIKV, respectively. Viral load in plasma was determined by qRT-PCR and FFA on Vero cells 3 days after each inoculation. Body weight was monitored every 2 days post-infection. After observation of any dead pup or weight loss of ≥ 20 or ≥ 10% in association with one suffering symptom, the whole litter was immediately euthanized. Spleen and brain were collected from euthanized pups and subjected to RNA extraction for qRT-PCR.

### Cell Infections

Cells were infected as previously described (Hubert et al., 2019) with two Asian strains of ZIKV, one from Pacific Islands (H/PF13) and one from South America (Brazil/2016). Different multiplicities of infection (MOI) were used as indicated in each experiment. At several time points, infection was quantified by immunofluorescence and flow cytometry after staining of ZIKV envelope protein (E), as described below. The viral load in the supernatant was measured by qRT-PCR and foci forming assay.

For intestinal epithelium infection and crossing experiments, various amounts [10^4^, 10^5^, and 10^6^ foci forming units (FFU)] of the American (Brazil/2016) strain of ZIKV were added in the apical compartment of differentiated Caco-2 tight monolayers (14 to 28 days in culture). Infection was performed as described above. Monolayer tightness was followed during all the experiment, and viral production was analyzed at different times post-infection.

### Immunofluorescence

To assess ZIKV infection in cells, supernatant was removed at the indicated times and cells were rinsed in PBS. Then, cells were fixated in a 4% paraformaldehyde (PFA) solution for 15 min at RT and permeabilized with PBS – 0.2% Triton for 5 min at RT. Then, ZIKV envelope protein (E) staining was performed overnight at 4°C using a primary mouse anti-E antibody (4G2), and for 1 h at RT with a secondary Alexa Fluor 488-coupled goat anti-mouse antibody (Life technologies). Finally, cells were mounted in Fluoromount G – DAPI (SouthernBiotech) and imaged on a fluorescence microscope (EVOS FL, Life Technologies).

Presence of tight junctions in Caco-2 monolayers was assessed by zonula occludens-1 (ZO-1) immunostaining. Cells were fixed in PBS – 80% methanol (Sigma) for 15 min at RT and permeabilized with PBS – 0.2% Triton for 5 min at RT. Non-specific sites were blocked with PBS – 5% BSA for 30 min at RT. ZO-1 was stained by incubating cells with a polyclonal rabbit anti-ZO-1 (Invitrogen) overnight at 4°C as primary antibody, and an Alexa Fluor 546-coupled donkey anti-rabbit (Invitrogen) for 1 h at RT as secondary antibody, using the same buffers as previously (Hubert et al., 2019). Finally, cells were mounted in Fluoromount G – DAPI and imaged on the same fluorescence microscope as above. Cells were washed twice with PBS between each step.

### Flow Cytometry

To quantify ZIKV-infected cells, cells were rinsed in PBS and dissociated by trypsinization (Gibco, by Life Technologies). Intracellular staining of envelope protein E was performed as previously described (Hubert et al., 2019). Briefly, detached and dissociated cells were fixated in a 4% paraformaldehyde (PFA) solution for 15 min at RT and permeabilized with PBS – 0.2% Triton for 5 min at RT. Then, ZIKV envelope protein (E) staining was performed using a primary mouse anti-E antibody (4G2) for 20 min at RT, and a secondary Alexa Fluor 488-coupled goat anti-mouse antibody (Life technologies) for 20 min at RT. Data were acquired by fluorescence activating cell sorting (FACS) using Gallios and CytoFLEX Beckman Coulter cytometers, and analysis was performed using FlowJo 10.0.8r1 software.

### qRT-PCR

To quantify ZIKV viral production at different times post-infection, cell supernatants were harvested and viral RNA was extracted as described previously (Hubert et al., 2019). For *in vivo* experiments, blood samples were harvested in EDTA-coated tubes (Sigma) and centrifuged for 15 min at 2000 *g*. Plasma were collected and extracellular RNA was extracted following the manufacturer’s protocol (QIAmp Viral RNA Mini Kit, Qiagen). Neonatal mice organs were dissociated by strong agitation (2 cycles of 4000 rpm for 20 s) in the presence of 2.8 mm ceramic beads (Omni International) and RNA extractions were performed using Trizol (Ambion, by Life Technologies). Reverse transcription was performed as previously described (Hubert et al., 2019) by using random hexamers and the SuperScript IV First-Strand cDNA Synthesis or Maxima H Minus Reverse-Transcriptase kits (Invitrogen). Quantitative PCR was performed as previously described (Hubert et al., 2019) by using MasterMix (iTaq Universal SYBR Green Supermix, Biorad), 500 nM of each ZIKV NS5-specific primers (Forward: 5′ – AAR TAC ACA TAC CAR AAC AAA GTG GT – 3′; Reverse: 5′ – TCC RCT CCC YCT YTG GTC TTG – 3′), and with the following program: 10 min/95°C followed by 40 three-step cycles of 15 s/95°C; 20 s/60°C; 30 s/72°C (Mastercycler Eppendorf Realplex). Quantification analysis was performed using a standard curve of ZIKV-encoding plasmid.

### Ethics Statement

Experiences on animals were performed in compliance with French and European regulations on care and protection of laboratory animals (EC Directive 2010/63, French Law 2013-118, February 6th, 2013). All these experiments were approved by the Ethics Committee #89 and registered by the French “Ministère de l’Enseignement supérieur, de la Recherche et de l’Innovation” under the reference “APAFIS#9594-2017041412342250v3” (date of approval: 2018 June 11th). Usage of genetically modified mice (A129) was approved by the institutional instances and the French “Ministère de l’Enseignement supérieur, de la Recherche et de l’Innovation” under the reference no. 2194 (date of approval: 2017 October 6th).

### Statistical Analysis

All statistical analyses were performed using Prism 7 software. All data were representative of three independent experiments except when mentioned in the legend, and presented as mean ± SD. Each test was detailed in Figure legends.

## Results

### ZIKV-Infected Dams Transfer ZIKV Infection to Suckling Pups During Breastfeeding

**FIGURE 1:**
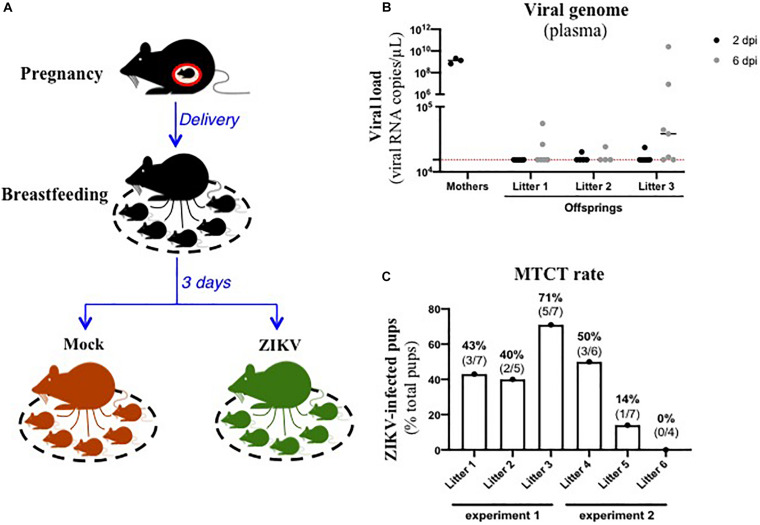
ZIKV-infected dams transfer ZIKV infection to suckling pups during breastfeeding. **(A)** Three days after delivery, A129 female mice were exposed to 2.8 × 10^6^ FFU of the American (Brazil/2016) strain of ZIKV via intraperitoneal route. Two and six days after mother’s infection, blood was sampled in lactating dams and suckling pups to evaluate viremia. **(B)** Viremia was measured by qRT-PCR in plasma of suckling pups from mock-treated mothers (black symbols “mothers”) or ZIKV-infected dams at 2 days (black symbols) and 6 days (gray symbols) after mother’s infection. The red dashed line represents the limit of specificity, evaluated from mock-treated mice. **(C)** Efficiency of mother-to-child transmission (MTCT) of ZIKV via breastfeeding was estimated and expressed as a percentage of ZIKV-infected suckling pups over total pups from the same litter. This graph recapitulates results obtained from two independent experiments (experiment 1 and experiment 2, with 3 litters/experiment). Results of the other experiment (experiment 2) are shown in Supplementary Figure S1.

In order to assess the existence of breastfeeding as a mode of transmission of ZIKV, we modelized the whole process of viral MTCT in A129 mice. Thus, we mated A129 males and females to obtain pregnant female mice. Three days after delivery, lactating female mice were intraperitoneally inoculated with the American (Brazil/2016) strain of ZIKV (Figure 1A). As shown in Figure 1B, all ZIKV-exposed mothers developed a high plasma viral load as early as 2 dpi (1.4 × 10^9^ ± 4.7 × 10^8^ viral RNA copies/μL plasma), confirming their infection status. Viremia of suckling pups was monitored for 6 days after mother’s infection. Interestingly, 10/19 suckling pups breastfed from ZIKV-infected mothers became infected between 2 and 6 days after mother’s infection, with plasma viral load ranging from 1.7 × 10^4^ to 2.0 × 10^10^ viral RNA copies/μL (Figure 1B). Efficiency of ZIKV MTCT during breastfeeding was assessed from two independent experiments (Figure 1 and Supplementary Figure S1A). It varied according to the litter and amounted to approximatively 39% (14/36 pups) (Figure 1C). Independently of ZIKV infection in pups, a growth arrest was observed at day 2 in all suckling pups breastfed from ZIKV-infected mothers compared to pups breastfed from mock-treated mothers (Supplementary Figure S1B), probably due to the maternal infection and health status. Altogether, these data suggest that ZIKV can be transmitted from lactating mothers to suckling pups during breastfeeding.

### ZIKV Infects Neonatal Mice After Oral Exposure

**FIGURE 2:**
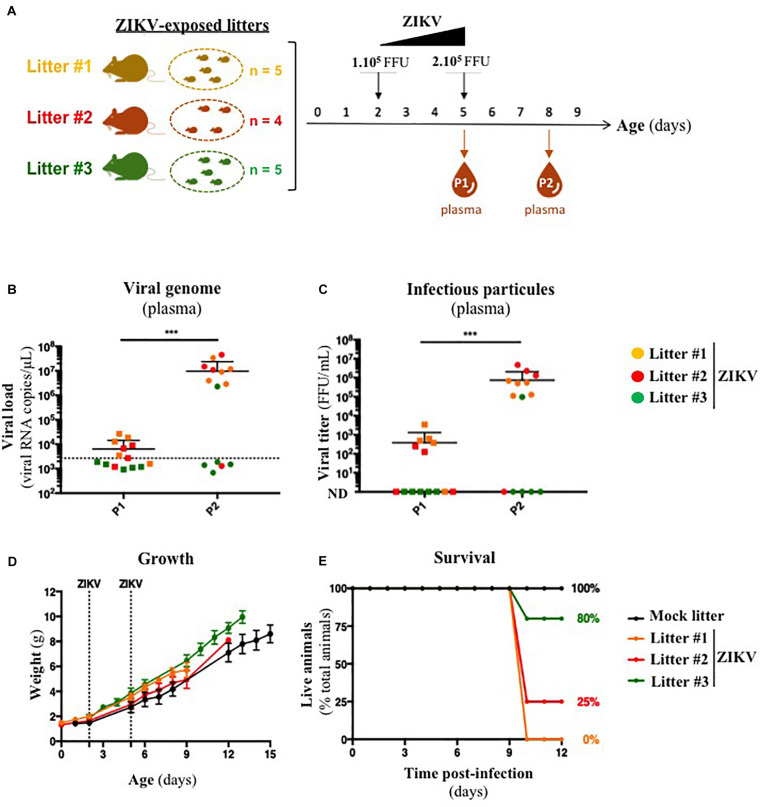
A129 offsprings are susceptible to ZIKV infection via oral route. **(A)** Three litters of four to five A129 pups (litter 1, orange; litter 2, red; litter 3, green) were orally exposed to ZIKV (Brazil/2016). Two successive doses, 1 × 10^5^ FFU and 2 × 10^5^ FFU, were administrated 2 and 5 days after birth, respectively. Plasma was sampled at 3 days after each administration (P1 and P2, for “Plasma 1” and “Plasma 2”) to measure plasmatic viral load. Viral load was measured in plasma by qRT-PCR **(B)** and foci forming assay on Vero cells **(C)**. The dotted line in **(B)** shows the limit of specificity, evaluated from mock-treated mice. ND in **(C)** means that the viral titer was non-detectable. Statistical test: logarithmic transformation coupled to paired one-way ANOVA (***p < 0.0005). **(D)** Body weight of offsprings was routinely monitored and growth curves were represented. **(E)** The survival rate was defined as a percentage of living animals per group. All results were expressed as mean ± standard deviation and representative of three independent experiments.

**FIGURE 3:**
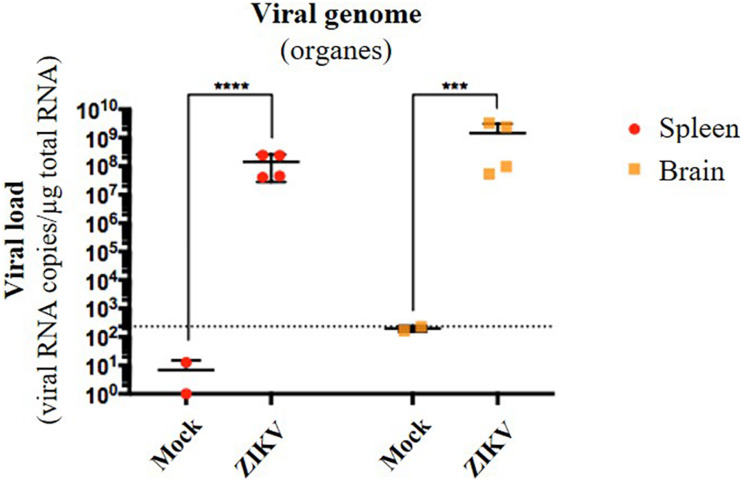
Oral infection of neonatal mice with ZIKV is associated to viral neuroinvasion. Seven pups from another independent littermate were orally exposed at 2 and 5 days-old to 1 × 10^5^ FFU and 2 × 10^5^ FFU of ZIKV (Brazil/2016), respectively. Two mock-treated mice were used as negative controls. At 6 days after the first administration, the four surviving pups were euthanized and organs were collected. RNA was extracted from the whole spleen and brain, and viral burden was measured by qRT-PCR using NS5-specific primers. The dotted line shows the limit of specificity, evaluated from mock-treated mice. Statistical test: logarithmic transformation coupled to unpaired *t*-test (***p < 0.0005; ****p < 0.0001). All results were expressed as mean ± standard deviation and representative of three independent experiments.

Several organs and/or tissues of the gastrointestinal and respiratory tracts could play a role as entry site for ZIKV during breastfeeding, such as tonsils, lungs, or the intestinal epithelium. Thus, we investigated the potential of ZIKV infectious particles to infect neonatal mice via the gastrointestinal tract. Although subcutaneous and intraperitoneal ZIKV infections of neonatal mice are well-characterized (Morrison and Diamond, 2017; Souza et al., 2018), oral infection has never been documented. Here, we orally infected neonatal mice with successive infectious doses of ZIKV in order to mimick the progressive viral absorption which could occur during breastfeeding. Three litters of 4–5 pups were inoculated via the oral route at 2 and 5 days-old by the American (Brazil/2016) strain of ZIKV. We assessed viremia 3 days after each oral inoculation by qRT-PCR and foci forming assay on Vero cells (Figure 2A). 5/5 pups became infected in the first litter (Supplementary Figures S2A,B, orange bars), 3/4 in the second (Supplementary Figures S2A,B, red bars) and 1/5 in the third (Supplementary Figures S2A,B, green bars). Globally, we showed that 64% of neonatal mice (9/14) were orally infected by ZIKV, whose plasma viremia ranged from 2.3 × 10^6^ to 4.6 × 10^7^ vRNA copies/μL at 6 days after the first inoculation (Figure 2B). In terms of infectious particles, plasma viremia increased from to 3.8 × 10^2^ to 7.5 × 10^5^ FFU/mL between 3 and 6 days after the first inoculation, respectively (Figure 2C). Although no significant deficit of growth was observed for ZIKV-exposed pups compared to mock-treated pups (Figure 2D), almost all infected neonates (7/9) died 9 to 10 days after the first inoculation (Figure 2E). The high mortality rate (78%; 7/9 infected pups) which is associated to oral infection prompted us to explore the dissemination profile of ZIKV in neonatal mice after this infection route. In another experiment, seven pups were orally exposed to the American (Brazil/2016) strain of ZIKV following the same infections scheme than previously. All of them (7/7) became infected without any significant body weight loss (data not shown). However, 3/7 pups died upon infection at 5 days after the first inoculation, confirming the high pathogenicity of ZIKV oral infection. The 4/7 other pups were euthanized the same day for organs collection and ZIKV genome was detected in spleen for all of them (Figure 3), confirming their infection status. Interestingly, ZIKV genome was also detected in the brain of all pups (4/4) at high levels (up to 1.5 × 10^9^ viral RNA copies/μg total RNA) (Figure 3). Taken together, these results demonstrate that ZIKV can orally be transmitted to neonatal mice, leading to high mortality.

### Human Intestinal Epithelial Cells Are Permissive to ZIKV Infection

**FIGURE 4:**
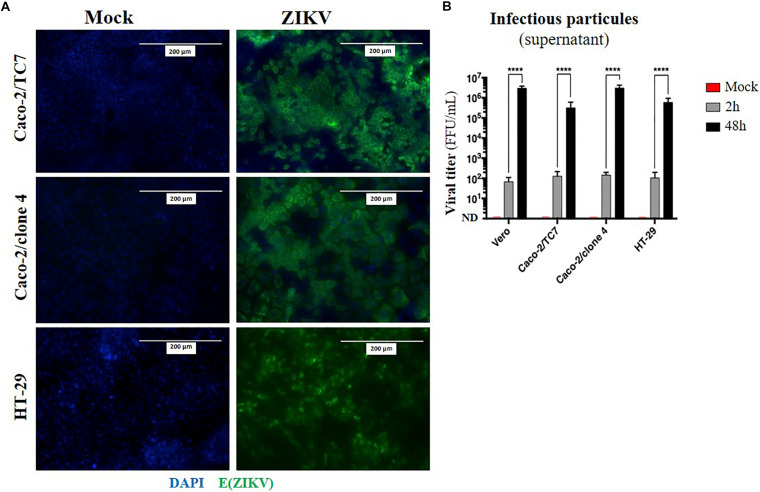
Human enterocyte-like cells are permissive to ZIKV infection. Enterocyte-like cells Caco-2 (clone TC7, clone 4) and HT-29 were infected by ZIKV (Brazil/2016) at MOI 1 for 48 h. **(A)** At 48 h post-infection, envelope protein of ZIKV [E(ZIKV)] expression was visualized by immunofluorescence using a pan-flavivirus antibody (4G2; green). Nuclei were stained with DAPI (blue). Bar scale: 200 μm. **(B)** Viral production of infectious particles in the supernatant was measured by foci forming assay at 2 and 48 h post-infection. Vero cells were used as a positive control for productive ZIKV infection. ND, not detectable. Statistical test: two-way ANOVA (****p < 0.0001). All results were expressed as mean ± standard deviation and representative of three independent experiments.

Given that oral inoculation of ZIKV in suckling pups leads to systemic infection and that viral particles need to cross an epithelium in the digestive tract to infect the host (tonsils, gut), we wondered whether the intestinal epithelium could serve as portal site for ZIKV oral infection in mice. To investigate whether ZIKV is able to infect a new host by crossing the intestinal epithelium, we firstly studied the permissivity of human intestinal epithelial cells to ZIKV infection. To this end, we infected two human adenocarcinoma-derived enterocyte-like cell lines (Caco-2/clone 4 or clone TC7, and HT-29) with the American (Brazil/2016) strain of ZIKV at MOI 1. As shown in Figure 4A, no staining of ZIKV envelope protein was observed by immunofluorescence in mock-treated cells. However, all ZIKV-exposed enterocyte-like cell lines were positive for ZIKV envelope protein. As demonstrated by the quantification of infectious particles in the supernatant, ZIKV infection of these cells results in a high viral production of infectious particles over time, resulting in similar viral loads than Vero cells (Figure 4B). Similar results were observed by infecting Caco-2 cells with the Asian (H/PF13) strain of ZIKV (Supplementary Figure S3). Importantly, we observed a dose-dependent staining of ZIKV envelope protein at MOI 0.1 and 1 with the presence of foci of infection (Supplementary Figure S3A, white squares) at MOI 0.1 which is overcome at MOI 1, suggesting a viral propagation of ZIKV from an infected cell to adjacent cells. At 48 h post-infection with the Asian (H/PF13) strain, we found that infection of Caco-2 cells concerned about 30% of cells at MOI 1 (Supplementary Figures S3B,C), indicating the important sensitivity of these cells to ZIKV infection. Finally, we confirmed that enterocyte-like Caco-2 cells were also productively infected by the Asian (H/PF13) strain of ZIKV in a dose-dependent manner (data not shown). Thus, we demonstrate that the different clones of human enterocyte-like cells are permissive to infection with different strains of ZIKV.

### ZIKV Infects and Crosses an *in vitro* Model of Intestinal Epithelium Without Causing Any Loss of Barrier Function

**FIGURE 5:**
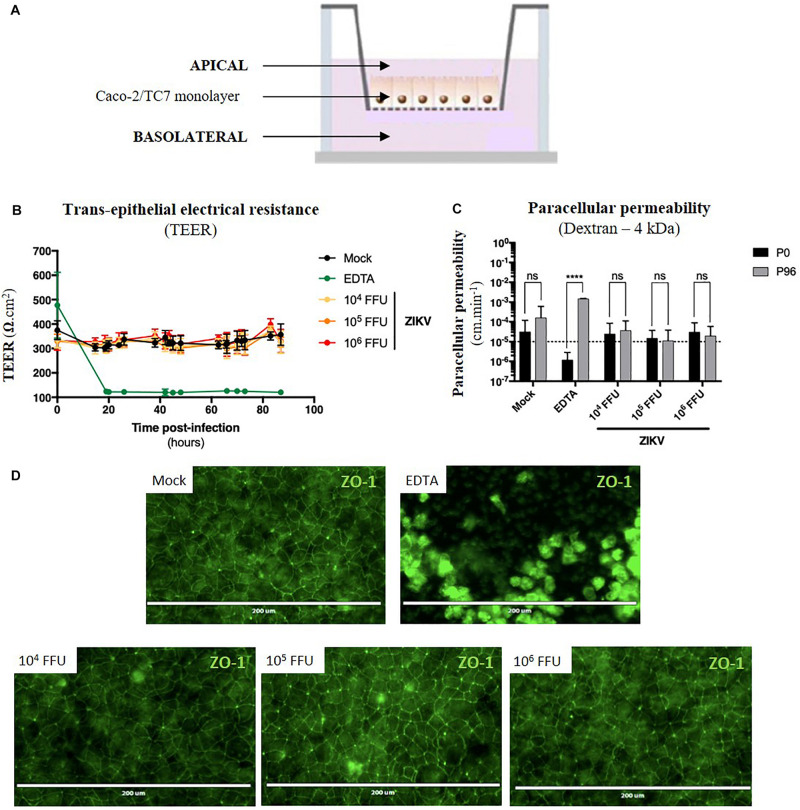
ZIKV infection of the *in vitro* model of intestinal epithelium does not lead to any loss of barrier function. **(A)** 2.5 × 10^4^ Caco-2/TC7 cells were seeded onto Transwell^®^ inserts (porosity: 3 μm) and cultured for 21 days for differentiation, resulting in the formation of a tight and polarized intestinal epithelial monolayer delimiting apical (upper chamber) and basolateral (lower chamber) compartments. 10^4^, 10^5^, and 10^6^ FFU of ZIKV (Brazil/2016) were apically added onto tight TC7 monolayers inserts and barrier integrity was monitored over time. As positive control of tightness alteration, Caco-2/TC7 monolayers were treated with 12.5 mM of EDTA, a cations-chelating agent. **(B)** *Trans*-epithelial electrical resistance (TEER) of ZIKV-exposed Caco-2/TC7 monolayers (yellow, orange, and red lines) was followed over time and was compared to TEER of mock-treated (black line) and EDTA-treated (green line) Caco-2/TC7 monolayers. Results were expressed as Ω.cm^2^. **(C)** Paracellular permeability “P” to dextran (4 kDa) was measured before infection (P0) and 90 h post-infection (P90). Statistical test: two-way ANOVA (****p < 0.0001). **(D)** Tight junctions’ integrity was visualized at 90 h post-infection by immunofluorescence after ZO-1 staining. Bar scale: 200 μm. All results were expressed as mean ± standard deviation and representative of three independent experiments.

As ZIKV infects human enterocyte-like cells, we wondered whether ZIKV could be basolaterally released after apical infection of fully differentiated enterocyte-like cells. To this end, we used a previously reported *in vitro* model of intestinal epithelium by seeding Caco-2 cells onto Transwell inserts which form tight intestinal monolayers after 14 to 21 days of culture, delimiting apical and basolateral compartments (Figure 5A). During cell proliferation and differentiation, *trans*-epithelial electrical resistance (TEER) of Caco-2 monolayers increased until reaching a maximal TEER of 250–300 Ω.cm^2^ (Supplementary Figure S4A), corresponding to a tight and fully differentiated intestinal epithelium (Martin-Latil et al., 2012). As model validation, structural features of a differentiated intestinal epithelium were observed by transmission electron microscopy such as microvilli (Supplementary Figure S4B, black arrows) and tight junctions (Supplementary Figure S4B, white arrows). Tight junctions’ formation could also be visualized after ZO-1 protein staining, confirming the tightness of the intestinal barriers (Supplementary Figure S4C). From a functional point-of-view, tightness-reflecting paracellular permeability of the model was evaluated by measuring the apical-to-basolateral crossing of a small dextran polymer (4 kDa) coupled to FITC (Supplementary Figure S4D). Paracellular permeability values less than 10^–5^cm.min^–1^ were required to consider Caco-2 monolayers as impermeable.

For the experiment, different infectious doses of the American (Brazil/2016) strain of ZIKV were apically added onto tight monolayers and evolution of monolayer tightness upon infection was followed for 90 h. As a positive control of alteration, Caco-2 monolayers were treated with 12.5 mM of a cations-chelating agent, EDTA, which disrupts tight junctions integrity. As shown in Figure 5B, EDTA treatment of Caco-2 monolayers resulted in a drastic drop of the TEER to 100 Ω.cm^–2^ (Figure 5B, green line), demonstrating the link between TEER and monolayer tightness. Interestingly, TEER of ZIKV-exposed Caco-2 monolayers did not diminish over time after infection (Figure 5B, yellow, orange, red lines), independently of the viral dose. Although paracellular permeability of EDTA-treated Caco-2 monolayers was increased by a factor 1000, no significant increase in paracellular permeability to dextran (4 kDa) occurred in monolayers exposed or not to ZIKV, regardless of the amount of viral particles added to the apical pole (Figure 5C). Moreover, we did not observe any difference of ZO-1 staining between mock-treated and ZIKV-infected monolayers at both 48 h (data not shown) and 90 h post-infection (Figure 5D), confirming that tight junctions were maintained upon infection. Thus, ZIKV infection of an *in vitro* model of intestinal epithelium does not result in alteration of its barrier function.

**FIGURE 6:**
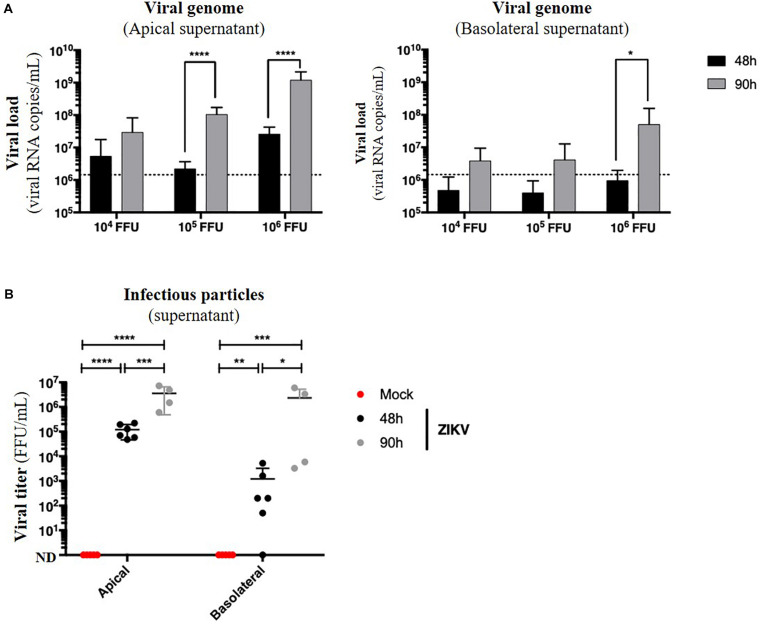
ZIKV infects and crosses the *in vitro* model of intestinal epithelium. 10^4^, 10^5^, and 10^6^ FFU of ZIKV (Brazil/2016) were apically added onto tight TC7 monolayers cultivated onto Transwell^®^ inserts. Crossing of ZIKV was evaluated by quantifying ZIKV RNA levels in both apical and basolateral compartments by qRT-PCR at 48 and 90 h post-infection using NS5-specific primers **(A)** and by titration on Vero cells. ND, not detectable. **(B)** The dotted line in **(A)** shows the limit of specificity, evaluated from the supernatant of mock-treated Caco-2/TC7 monolayers. Statistical test: *t*-test after logarithmic transformation (*p < 0.05; **p < 0.005; ***p < 0.0005; ****p < 0.0001). All results were expressed as mean ± standard deviation and representative of three independent experiments.

Because no monolayer tightness alteration was observed, we wondered whether ZIKV infection of Caco-2 monolayers resulted in basolateral production of infectious particles. Apical and basolateral supernatants of impermeable ZIKV-infected monolayers were harvested at different times post-infection, and the viral load was measured. In both apical and basolateral compartments, we observed a 10- to 100-fold increasing of the extracellular viral RNA copies levels between 48 and 90 h post-infection, demonstrating a viral production on both sides of the epithelium (Figure 6A). After comparison of viral loads of supernatants from monolayers exposed to different doses of ZIKV, we also observed a dose-dependent effect of this viral production (Figure 6A) as well as a significant production of infectious particles in the basolateral supernatant between 48 h (0 to 5.2 × 10^3^ FFU/mL) and 90 h post-infection (2.3 × 10^6^ FFU/mL) (Figure 6B).

Taken together, these results demonstrate that ZIKV is able to infect a polarized model of tight intestinal epithelium without interfering with barrier integrity, and subsequently be released at the basolateral side.

## Discussion

During the past two decades, ZIKV emerged in the Pacific islands (Musso et al., 2014) and Latin America (Baud et al., 2017). These emergences focused the interest of numerous studies, providing new findings concerning non-vector borne transmission of ZIKV. Especially during the American outbreak, clinical and experimental data permitted to recognize sexual and transplacental mother-to-child transmissions of ZIKV (D’Ortenzio et al., 2016; Rasmussen et al., 2016). However, since ZIKV was detected in many body fluids of infected persons, other modes of human-to-human transmission need to be considered (Grischott et al., 2016). Particularly, high viral loads of ZIKV infectious particles were detected in both colostrum and mature breast milk (Sotelo et al., 2017; Mann et al., 2018), and the first confirmed ZIKV MTCT via breastfeeding was reported in 2018 (Blohm et al., 2017, 2018). Here, we achieved the first study to assess an experimental proof-of-concept of the existence of breastfeeding as an uncommon mode of transmission of a Brazilian strain of ZIKV, and proposed the intestinal epithelium as a potential entry site for ZIKV after oral exposure.

Although the exact nature of ZIKV in breast milk remains unknown, either cell-free, cell-associated, or as extracellular vesicle-cloaked virus clusters, breast milk of some infected mothers was shown to be infectious (Dupont-Rouzeyrol et al., 2016; Blohm et al., 2017; Cavalcanti et al., 2017; Sotelo et al., 2017). Several studies demonstrated that ZIKV viral particles remained infectious in fresh breast milk (Pfaender et al., 2017; Conzelmann et al., 2019) and that potential antiviral properties of breast milk were not sufficient to prevent ZIKV transmission to mice neonates (Pang et al., 2020). These observations led us wonder whether ZIKV could orally infect a suckling child during breastfeeding. Thus, we used a well-established murine model (A129) to investigate the potential oral susceptibility of neonate mice to ZIKV infection. Since ZIKV is able to inhibit IFN type I signaling in human cells but not in murine cells (Grant et al., 2016), mice that are deficient for type I interferons receptor, such as A129 mice, are commonly used to study virus-host interactions and recapitulate ZIKV pathophysiology observed in humans (multi-visceral dissemination, viral persistence in central nervous system and testes, viral excretion in body fluids, transplacental, and sexual transmissions,…). By exposing lactating dams to a Brazilian strain of ZIKV 3 days after parturition, we noted that 39% of suckling pups became infected. Interestingly, MTCT of another strain of ZIKV (Asian strain) by breastfeeding was recently highlighted (Pang et al., 2020). Because ZIKV had been previously demonstrated to be excreted in murine breast milk, transfer of infectious particles from ZIKV-infected dams to suckling pups via oral swallowing of contaminated breast milk is thus very probable. However, other excreted maternal fluids – such as urine and saliva – could also serve as a vehicle for ZIKV infectious particles transfer to suckling pups and cannot be excluded as a potential route of transmission (Barzon et al., 2016; Campos et al., 2016). Therefore, further studies need to be implemented to formally confirm MTCT of ZIKV through breastmilk, such as administration of breast milk from ZIKV-infected mothers to pups of uninfected mothers (housed in separate cages).

Following oral ingestion of infectious particles, several gastro-intestinal tract structures could allow ZIKV to enter the host. In macaques, Newman and colleagues demonstrated that close contacts between ZIKV and tonsils result in systemic infection (Newman et al., 2017). Moreover, susceptibility of macaques to ZIKV infection after intragastric inoculation suggested other entry sites for ZIKV such as the intestinal epithelium (Deng et al., 2017). Indeed, ZIKV was recently found to be transmissible via intra-anal route through the anorectal mucosa (Li et al., 2018) after sexual intercourse, but no study explored the role of the small intestine in the context of oral exposure. Here, we found that ZIKV was efficiently able to be transmitted to A129 mouse offsprings via oral route, leading to a systemic infection (64%) with a high associated mortality (78%). Although causes of mortality remain unknown, we could suspect neurological disorders since ZIKV genome was detected in the brain of ZIKV-infected pups (1.5 × 10^9^ viral RNA copies/μg total RNA). Although these first observations raise interesting questions about the pathogenicity of ZIKV after oral infection, immunohistochemistry-based detection of ZIKV antigens would permit to specify this neurotropism. Moreover, oral infections were assessed by inoculating cell culture supernatants, we acknowledge that multiple biologically active molecules found in breast milk are not represented and could also have an impact on oral infection efficiency. In further studies, addition of human fresh breast milk or several of these breast milk components (triglycerides, free fatty acids, lactoferrin, lysozyme, IgAs, immune cells) would help to better understand mother’s factors which positively or negatively modulate oral transmission of ZIKV. In other experiments, we confirmed that a single intragastric exposition of suckling pups to ZIKV led to an efficient systemic infection, strengthening the potential role of the intestinal epithelium as ZIKV entry site.

Given that several orally-transmitted viruses enter the host by crossing the intestinal epithelium, either by infection of enterocytes (Rotavirus; Ramig, 2004) or by transcytosis via enterocytes and/or M cells (HTLV-1; Martin-Latil et al., 2012), HIV (Bomsel, 1997), Poliovirus (Ouzilou et al., 2002), Norovirus (Gonzalez-Hernandez et al., 2013), we wondered whether ZIKV could use one of these strategies to cross the intestinal barrier and infect the host. As demonstrated by Chan et al. (2016), we firstly confirmed that several human enterocyte cell lines were permissive to ZIKV infection. However, interactions between ZIKV and polarized intestinal cells had been investigated in a unique study for only 48 h (Tamhankar and Patterson, 2019). Interestingly, we showed that addition of different infectious loads to the apical pole of tight and polarized monolayers of differentiated Caco-2/TC7 cells resulted in production of infectious viral particles by both apical and basolateral poles from 48 to 90 h post-infection. In addition, this infection is not accompanied by any significant alteration in the integrity of the tight junctions and the permeability of the monolayers, demonstrating that ZIKV is able to infect and cross an *in vitro* model of intestinal epithelium without altering its barrier function. Our data suggest that ZIKV infection of intestinal epithelial cells is not associated to consequent cytopathic effects which compromises the intestinal barrier function during the whole lifespan of the human intestinal epithelium (3–5 days). Because ZIKV infectious entities in breast milk are unknown, future research should emphasize on the possibility of milk vesicle-cloaked virus clusters to transit through the gastrointestinal tract and achieve a high multiplicity of infection at the intestinal site (Santiana et al., 2018), or the possibility of milk ZIKV-infected cells to transmigrate through the intestinal epithelium (Molès et al., 2018; Ayala-Nunez et al., 2019; de Carvalho et al., 2019). Especially, it was shown that ZIKV free virions were able to cross an *in vitro* model of blood-testicular barrier without altering its barrier function, but the presence of ZIKV-infected macrophages supernatant, which contains various inflammatory cytokinic mediators (TNF-α, IL-1α, IL-8), led to its disruption (Siemann et al., 2017). Thus, the presence of ZIKV-infected cells and inflammatory mediators at the apical and/or basolateral poles of the intestinal epithelium could also induce an increase in paracellular permeability of the intestinal epithelium, facilitating further crossing by free virions. Finally, the intestinal barrier is defined as an apical-to-basolateral stack of four main barriers: the microbiota (microbiological barrier), the mucus layers and associated antimicrobial peptides (chemical barrier), the tight intestinal epithelium (physical barrier), and the *lamina propria* and associated immune cells (immunological barrier). Thus, the effectiveness of the crossing of the intestinal epithelium by ZIKV could also be modulated by many factors of those barriers such as bacteria and associated prebiotics, mucins, antimicrobial peptides, or secretory IgA. A more in-depth study on the interactions between ZIKV and the various actors of the intestinal barrier as well as the mechanisms of epithelial crossing could therefore interesting to implement.

At the present time, the official recommendation of the World Health Organization (WHO) claims that “the benefits of breastfeeding for the infant and mother outweigh any potential risk of ZIKV transmission through breast milk” (WHO, 2019). Indeed, breastfeeding is well-recognized as protective against several pathologies (sudden infant death syndrome, necrotizing enterocolitis, gastro-intestinal and respiratory infections) and could reduce the pathogenicity of other diseases such as asthma, auto-immune diseases (type 1 diabetes), or obesity and associated diseases (cardiovascular diseases, hypercholesterolemia, hypertension…). These beneficial effects are mainly due to the diversity and abundance of nutritive (proteins, lipids, lactose, vitamins), trophic (growth factors), and immunological (IgA, immune cells) components of the breastmilk, as well as its antimicrobial activity against numerous pathogens. However, due to the asymptomatic or non-specific clinical profile of most ZIKV infections, underdiagnoses made difficult to estimate the real prevalence of ZIKV in populations, and ZIKV excretion in body fluids was not systematically researched. As a consequence, no sufficient studies reported ZIKV infectious particles and/or genome in breast milk (Besnard et al., 2014; Dupont-Rouzeyrol et al., 2016; Blohm et al., 2017; Cavalcanti et al., 2017; Colt et al., 2017; Sotelo et al., 2017; Giovanetti et al., 2018), making breastfeeding an underappreciated route of ZIKV transmission. Thus, several epidemiological and experimental approaches are necessary to provide the needed evidence to confirm its existence. From an epidemiological point of view, the systematic search for the viral genome and/or infectious particles in the breast milk of infected lactating women should be carried out in endemic areas and epidemic situations, as well as the diagnosis of infants breastfed by an infected woman. Contrary to other milk-borne viruses – such as HIV-1 or HTLV-1 – the abundance of the vector in those areas and situations makes vector-borne transmissions a major concern and the formal prove of ZIKV milk-borne transmission to newborns remains difficult to assess yet. Therefore, an experimental proof of concept of mother-to-child transmission of ZIKV during breastfeeding is essential and must be carried out in several ZIKV-susceptible animal models (mice, guinea pigs, monkeys). In this study, we assessed an experimental reconstitution of the whole process of ZIKV MTCT during breastfeeding in a mouse model and provide an additional evidence to the pre-existing converging data (previously listed in introduction) supporting the existence of ZIKV MTCT during breastfeeding. As a consequence, this study could support further dedicated studies to several aspects of the breastfeeding-related MTCT of ZIKV. These future studies will be necessary to assess the potential risk linked to ZIKV transmission by breastfeeding, while keeping in mind the numerous benefits of breastfeeding, especially in low- and middle-income countries (LMIC).

In summary, we provided new insights concerning the mother-to-child transmission of ZIKV by breastfeeding. First, we assessed an experimental proof-of-concept of the existence of mother-to-child transmission of ZIKV during breastfeeding. Second, we demonstrated that neonatal mice were susceptible to ZIKV infection after intragastric inoculation, supporting previous reports of ZIKV oral transmission in animals and reinforcing the interest to explore this mode of transmission. Third, we considered the gut as a possible entry site of ZIKV following ingestion as we demonstrated that ZIKV infected and crossed an *in vitro* model of intestinal epithelium without altering its barrier function. Taken together, these results permit to reconsider the plausibility of mother-to-child transmission of ZIKV during breastfeeding, which remains actually underappreciated, and provide new perspectives to investigate.

## Data Availability Statement

All datasets generated for this study are included in the article/Supplementary Material.

## Ethics Statement

The animal study was reviewed and approved by the Ethics Committee #89 and registered by the French “Ministère de l’Enseignement supérieur, de la Recherche et de l’Innovation” under the reference “APAFIS#9594-2017041412342250v3” (date of approval: 2018 June 11th).

## Author Contributions

MH: conceptualization, methodology, formal analysis, data curation, validation, investigation, writing-original draft preparation, writing-review and editing, and visualization. PJ and PR: resources. JB-G: methodology (transmission electron microscopy experiments). AG: writing-review and editing and supervision. P-EC: conceptualization, methodology, validation, writing-review and editing, supervision, visualization, project administration, and funding acquisition. AV: conceptualization, methodology, validation, writing-review and editing, supervision, visualization, project administration, and funding acquisition. All authors contributed to the article and approved the submitted version.

## Conflict of Interest

The authors declare that the research was conducted in the absence of any commercial or financial relationships that could be construed as a potential conflict of interest.

## Supplementary Material

Supplementary Figure 1 ZIKV-infected dams transfer ZIKV infection to suckling pups during breastfeeding. **(A)** Three days after delivery, A129 female mice were exposed to 2.8 × 10^6^ FFU of the American (Brazil/2016) strain of ZIKV via intraperitoneal route. Two and six days after mother’s infection, blood was sampled in lactating dams and suckling pups to evaluate viremia. Viremia was measured by qRT-PCR in plasma of lactating dams (green symbols) and suckling offsprings (orange, red, and brown symbols). Viremia of offsprings breastfed from mock-treated mothers was null and was not represented. **(B)** Body weight of suckling pups offsprings was routinely monitored and growth curves were represented. All results were expressed as mean ± standard deviation and representative of two independent experiments. The other experiment is shown in Figure 1.

Supplementary Figure 2 Oral infection of neonatal mice with ZIKV. Individual plasmatic viral load of each pup from the three litters shown in Figure 2 was represented as RNA copies/μL **(A)** or FFU/mL **(B)** of plasma.

Supplementary Figure 3 Dose-dependence of ZIKV infection in enterocyte-like cells. **(A)** Caco-2/TC7 cells were infected by ZIKV (H/PF13) at MOI 0.1 and 1 for 48 h. Envelope protein of ZIKV, E(ZIKV), was stained using a pan-flavivirus antibody (4G2; green). Nuclei were stained with DAPI (blue). White squares represent foci of infection. Bar scale: 400 μm. **(B)** E(ZIKV) was stained using a pan-flavivirus antibody (4G2; green), and E(ZIKV)-positive cells were quantified by flow cytometry after infection at MOI 1. **(C)** Graphical representation of dot plots after FACS analysis examining E(ZIKV) expression. All results were expressed as mean ± standard deviation and representative of three independent experiments.

Supplementary Figure 4 Structural and functional caracterization of the *in vitro* model of intestinal epithelium. 2.5 × 10^4^ Caco-2/TC7 cells were seeded onto Transwell^®^ inserts (porosity: 3 μm) and cultured for 21 days for differentiation, resulting in the formation of a tight and polarized intestinal epithelial monolayer delimiting apical (upper chamber) and basolateral (lower chamber) compartments. **(A)** TEER of Caco-2/TC7 monolayers was measured over time and expressed as Ω.cm^2^. TEER of inserts alone was represented in grey. The red dotted line corresponds to the maximal value for an impermeable epithelium (published for these cells). **(B)** Structural features of polarized Caco-2/TC7 monolayers such as microvilli (black arrow) and tight junctions (white arrows) were observed by transmission electron microscopy. AP, apical pole; BL, basolateral pole. Bar scale: 1 μm. **(C)** Tight junctions were visualized by fluorescence microscopy after zonula occludens-1 (ZO-1) immunostaining. Bar scale: 200 μm. **(D)** Paracellular permeability of Caco-2/TC7 monolayers was evaluated after measurement of the apical-to-basolateral transport of dextran (4 kDa) coupled to fluoresceine isothiocyanate (FITC) by fluorimetry.
