# Supplementary figures and images for: Evidence That Zika Virus Is Transmitted by Breastfeeding to Newborn A129 (Ifnar1 Knock-Out) Mice and Is Able to Infect and Cross a Tight Monolayer of Human Intestinal Epithelial Cells
Source: Front Microbiol. 2020 Oct 22;11:524678. doi: 10.3389/fmicb.2020.524678 (PMC7649816; doi:10.3389/fmicb.2020.524678)

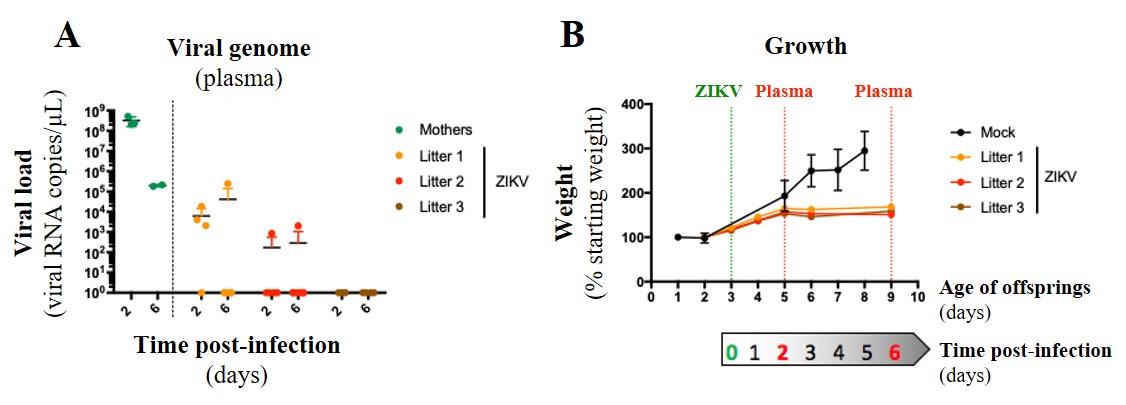

Supplement: Supplementary Figure 1 — ZIKV-infected dams transfer ZIKV infection to suckling pups during breastfeeding. (A) Three days after delivery, A129 female mice were exposed to 2.8 × 10^6^ FFU of the American (Brazil/2016) strain of ZIKV via intraperitoneal route. Two and six days after mother’s infection, blood was sampled in lactating dams and suckling pups to evaluate viremia. Viremia was measured by qRT-PCR in plasma of lactating dams (green symbols) and suckling offsprings (orange, red, and brown symbols). Viremia of offsprings breastfed from mock-treated mothers was null and was not represented. (B) Body weight of suckling pups offsprings was routinely monitored and growth curves were represented. All results were expressed as mean ± standard deviation and representative of two independent experiments. The other experiment is shown in Figure 1. [file Image_1.JPEG]

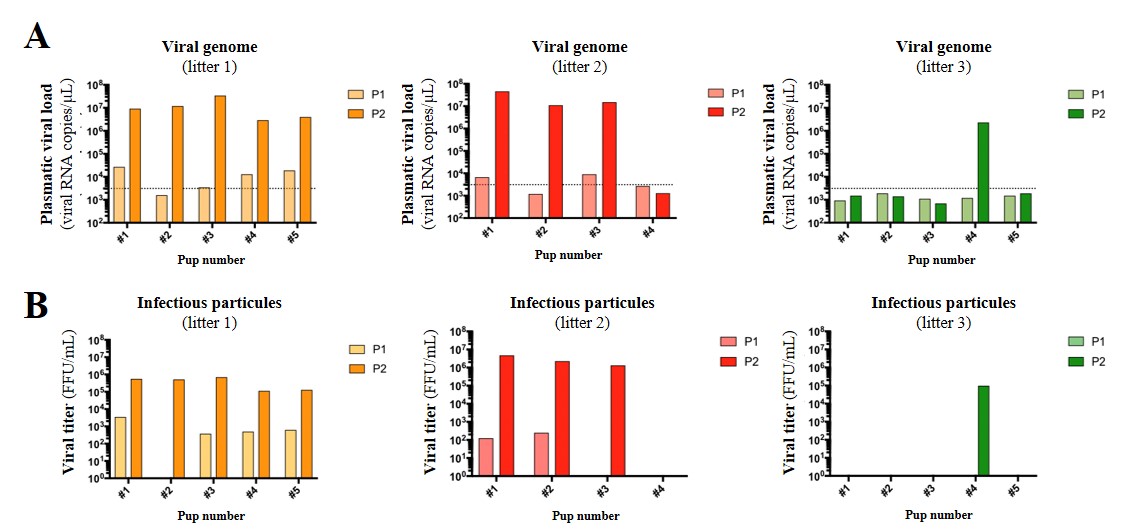

Supplement: Supplementary Figure 2 — Oral infection of neonatal mice with ZIKV. Individual plasmatic viral load of each pup from the three litters shown in Figure 2 was represented as RNA copies/μL (A) or FFU/mL (B) of plasma. [file Image_2.JPEG]

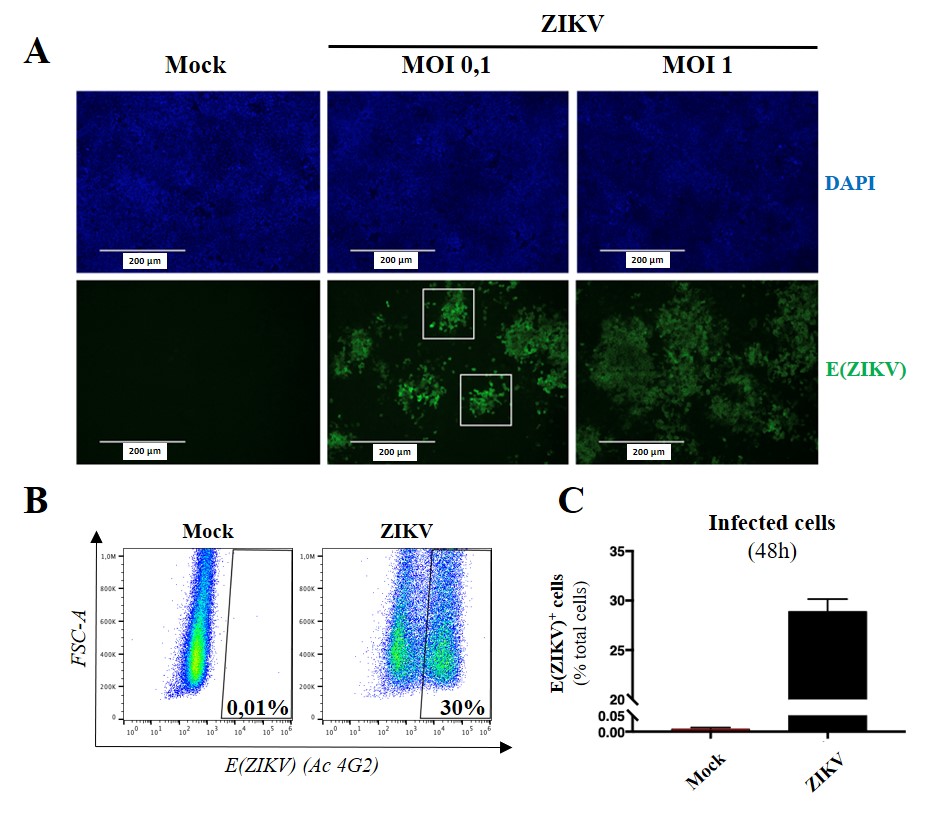

Supplement: Supplementary Figure 3 — Dose-dependence of ZIKV infection in enterocyte-like cells. (A) Caco-2/TC7 cells were infected by ZIKV (H/PF13) at MOI 0.1 and 1 for 48 h. Envelope protein of ZIKV, E(ZIKV), was stained using a pan-flavivirus antibody (4G2; green). Nuclei were stained with DAPI (blue). White squares represent foci of infection. Bar scale: 400 μm. (B) E(ZIKV) was stained using a pan-flavivirus antibody (4G2; green), and E(ZIKV)-positive cells were quantified by flow cytometry after infection at MOI 1. (C) Graphical representation of dot plots after FACS analysis examining E(ZIKV) expression. All results were expressed as mean ± standard deviation and representative of three independent experiments. [file Image_3.JPEG]

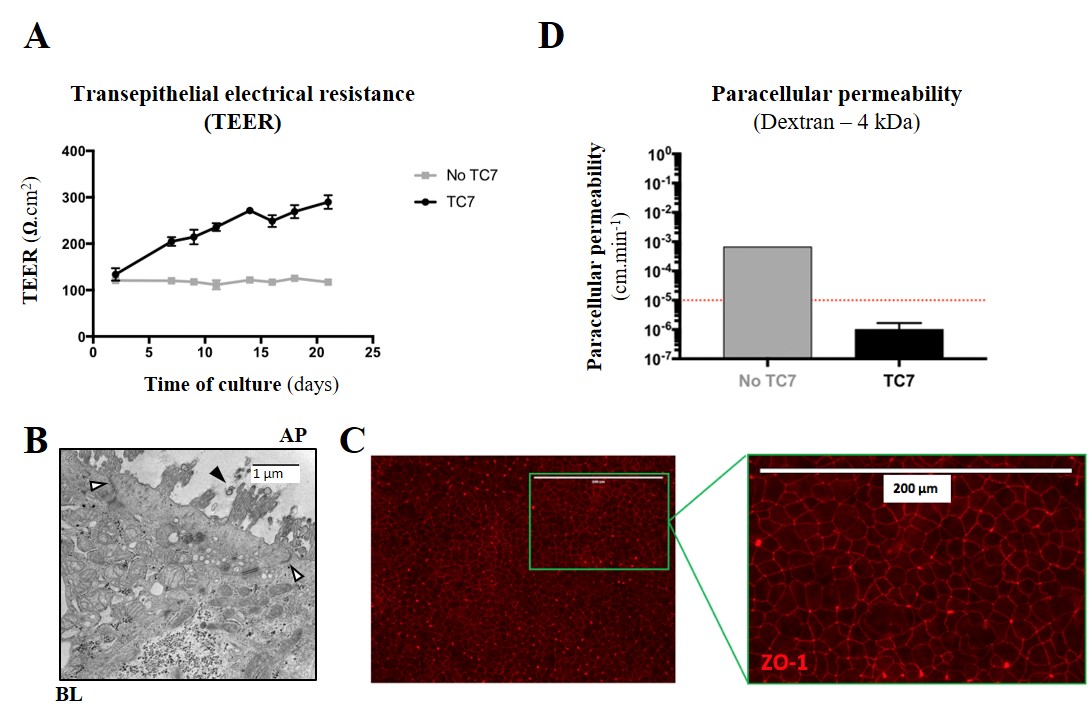

Supplement: Supplementary Figure 4 — Structural and functional caracterization of the in vitro model of intestinal epithelium. 2.5 × 10^4^ Caco-2/TC7 cells were seeded onto Transwell® inserts (porosity: 3 μm) and cultured for 21 days for differentiation, resulting in the formation of a tight and polarized intestinal epithelial monolayer delimiting apical (upper chamber) and basolateral (lower chamber) compartments. (A) TEER of Caco-2/TC7 monolayers was measured over time and expressed as Ω.cm2. TEER of inserts alone was represented in grey. The red dotted line corresponds to the maximal value for an impermeable epithelium (published for these cells). (B) Structural features of polarized Caco-2/TC7 monolayers such as microvilli (black arrow) and tight junctions (white arrows) were observed by transmission electron microscopy. AP, apical pole; BL, basolateral pole. Bar scale: 1 μm. (C) Tight junctions were visualized by fluorescence microscopy after zonula occludens-1 (ZO-1) immunostaining. Bar scale: 200 μm. (D) Paracellular permeability of Caco-2/TC7 monolayers was evaluated after measurement of the apical-to-basolateral transport of dextran (4 kDa) coupled to fluoresceine isothiocyanate (FITC) by fluorimetry. [file Image_4.JPEG]
